# Transamidation of Amides and Amidation of Esters by Selective N–C(O)/O–C(O) Cleavage Mediated by Air- and Moisture-Stable Half-Sandwich Nickel(II)–NHC Complexes

**DOI:** 10.3390/molecules26010188

**Published:** 2021-01-02

**Authors:** Jonathan Buchspies, Md. Mahbubur Rahman, Michal Szostak

**Affiliations:** Department of Chemistry, Rutgers University, 73 Warren Street, Newark, NJ 07102, USA; jonathan.buchspies@gmail.com (J.B.); rahman.mm11@gmail.com (M.M.R.)

**Keywords:** transamidation, twisted amides, NHCs, *N*-heterocyclic carbenes, nickel, Buchwald–Hartwig, amidation, cyclopentadienyl, nickel–NHCs, amide bonds, N–C activation, [CpNi(NHC)X]

## Abstract

The formation of amide bonds represents one of the most fundamental processes in organic synthesis. Transition-metal-catalyzed activation of acyclic twisted amides has emerged as an increasingly powerful platform in synthesis. Herein, we report the transamidation of *N*-activated twisted amides by selective N–C(O) cleavage mediated by air- and moisture-stable half-sandwich Ni(II)–NHC (NHC = N-heterocyclic carbenes) complexes. We demonstrate that the readily available cyclopentadienyl complex, [CpNi(IPr)Cl] (IPr = 1,3-bis(2,6-diisopropylphenyl)imidazol-2-ylidene), promotes highly selective transamidation of the N–C(O) bond in twisted N-Boc amides with non-nucleophilic anilines. The reaction provides access to secondary anilides via the non-conventional amide bond-forming pathway. Furthermore, the amidation of activated phenolic and unactivated methyl esters mediated by [CpNi(IPr)Cl] is reported. This study sets the stage for the broad utilization of well-defined, air- and moisture-stable Ni(II)–NHC complexes in catalytic amide bond-forming protocols by unconventional C(acyl)–N and C(acyl)–O bond cleavage reactions.

## 1. Introduction

**Figure 1:**
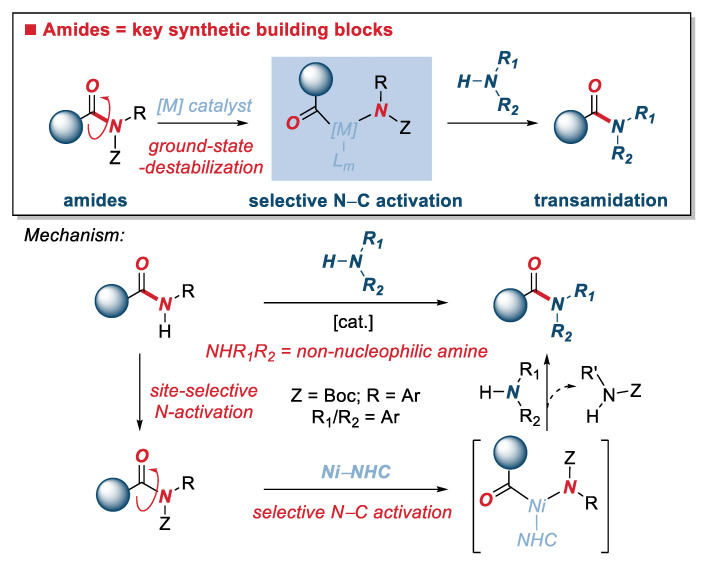
Transamidation of N-activated amides by selective N–C(O) cleavage.

The amide bond represents one of the most fundamental and important functional groups in organic synthesis [1,2,3]. It is estimated that amide bonds are the common structural motif in more than 75% of new pharmaceuticals, while new methods for the formation of amide bonds have been intensively investigated [4,5]. In this context, transamidation reactions represent a highly attractive, unconventional method for the synthesis of amide bonds by transforming a more reactive amide bond into a new, more thermodynamically stable amide counterpart [6,7,8,9,10]. In recent years, the selective activation of C(acyl)–N amide bonds has been achieved by the controlled metal insertion into the resonance activated bonds in twisted amides (i.e., non-planar amides) [11,12,13]. This general approach circumvents the low reactivity of amides resulting from n_N_→π^*^_C=O_ conjugation (resonance of 15–20 kcal/mol in planar amides), while providing a powerful platform for organic synthesis [14,15]. Transamidation reactions of twisted amide N–C(O) bonds have been achieved using well-defined Pd(II)–NHC catalysts as well as by using air-sensitive Ni(cod)_2_ in combination with NHC ligands [16,17,18,19,20,21]. These reactions provide a variety of novel methods for the synthesis of ubiquitous amide bonds and have been extended to catalytic amidation reactions of activated phenolic and unactivated methyl esters by O–C(O) cleavage [22,23,24,25]. In continuation of our studies on activation of amide bonds and organometallic catalysis, in this *Special Issue of Editorial Board* members of the *Organometallic Section* of Molecules, we report transamidation of *N*-activated amides by selective N–C(O) cleavage mediated by air- and moisture-stable half-sandwich Ni(II)–NHC (NHC = *N*-heterocyclic carbenes) complexes [26,27,28,29,30,31,32,33]. Most importantly, we demonstrate that readily available cyclopentadienyl complex extensively developed by Chetcuti, namely [CpNi(IPr)Cl] (IPr = 1,3-bis(2,6-diisopropylphenyl)imidazol-2-ylidene) [34,35,36,37,38,39,40,41,42,43], promotes highly selective transamidation of the N–C(O) bond in twisted N-Boc amides with non-nucleophilic anilines (Figure 1). The reaction provides access to secondary anilides via the non-conventional amide bond-forming pathway. Furthermore, the amidation of activated phenolic and unactivated methyl esters mediated by [CpNi(IPr)Cl] is reported. This study sets the stage for the broad utilization of well-defined, air- and moisture-stable Ni(II)–NHC complexes in catalytic amide bond-forming protocols by unconventional C(acyl)–N and C(acyl)–O bond cleavage reactions.

## 2. Results

**Figure 2:**
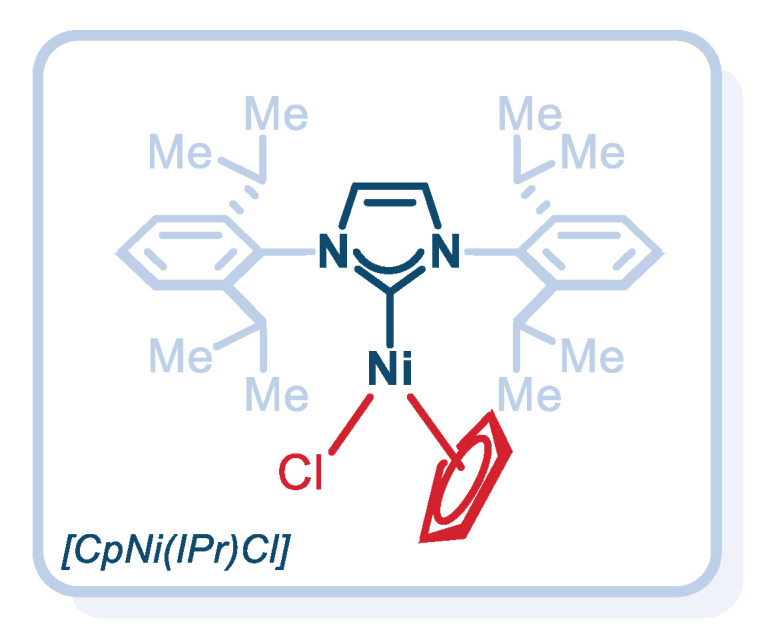
Structure of the air- and moisture-stable, well-defined, half-sandwich, cyclopentadienyl [CpNi(IPr)Cl] complex.

Although we have identified well-defined Pd(II)–NHC complexes for transamidation reactions of activated amides and esters [18,19,20,21], we have been investigating air- and moisture-stable Ni(II)–NHCs based on naturally more abundant Ni as 3d transition metal [26,27,28]. We were attracted to the well-defined, air- and moisture-stable, half-sandwich, cyclopentadienyl [CpNi(IPr)Cl] complex (Figure 2) owing to its ready availability, ease of handling and the potential to prepare more reactive cyclopentadienyl Ni(II)–NHC analogues [29,30,31,32,33]. Notably, [CpNi(IPr)Cl] has emerged as a highly attractive catalyst for several classes of cross-coupling reactions [29,30,31,32,33]; however, transamidations and amidation reactions using this well-defined catalyst have been elusive.

We initiated our studies by evaluating the reaction conditions for the [CpNi(IPr)Cl]-catalyzed transamidation of N-Boc activated amide **1a** with 4-methoxyaniline **2a** (Table 1). Of note, twisted N-Boc amides are readily prepared from the corresponding secondary amides by *N*-chemoselective *tert*-butoxycarbonylation. The *N*-carbamate activation permits for decreasing amidic resonance (RE, resonance energy, 7.2 kcal/mol), while providing a thermodynamic pathway for transamidation by rendering the leaving group non-nucleophilic [14,15]. After optimization, we have identified conditions for the transamidation in quantitative yield using [CpNi(IPr)Cl] (10 mol%) as a catalyst in the presence of K_2_CO_3_ as a base in toluene at 140 °C (Table 1, entry 1). We found that K_3_PO_4_ is also an effective base under these conditions (Table 1, entry 2). Furthermore, decreasing the catalyst loading to [CpNi(IPr)Cl] (5 mol%) resulted in lower conversions (Table 1, entries 3–4). Importantly, control reactions in the absence of the [CpNi(IPr)Cl] catalyst resulted in the recovery of the starting material, thus demonstrating that the catalyst is required for the reaction (Table 1, entries 5–6). Several other optimization conditions are worth noting (not shown): (1) lowering the reaction temperature resulted in significantly lower conversion (110 °C, 26%); (2) reactions at low catalyst loading resulted in low conversion (1 mol%, 13%).

With the optimized conditions in hand, the scope of the transamidation reaction catalyzed by the well-defined [CpNi(IPr)Cl] complex was examined with respect to the aniline component (Table 2). As shown, the reaction performed well using electron-donating (**3a**), para-substituted (**3b**), ortho-sterically hindered (**3c**), meta-substituted (**3d**), and electron-withdrawing (**3e**–**f**) anilines. It is worthwhile to note that the reaction efficiency decreased using electron-deficient nucleophiles. Furthermore, di-ortho-substituted anilines were unproductive substrates in the reaction, indicating excessive steric hindrance. 

Next, the scope of the reaction with respect to the amide group was evaluated (Table 3). As shown, primary and secondary alkyl amides (**3g**–**h**), electron-rich (**3i**–**j**) as well as electron-deficient (**3k**) aromatic amides underwent efficient transamidation under Ni–NHC catalysis. Furthermore, cinnamyl amide was found to be a suitable reaction partner for the transamidation (**3l**). Similar to the scope of anilines, steric hindrance on the amide component was not tolerated.

**Scheme 1:**
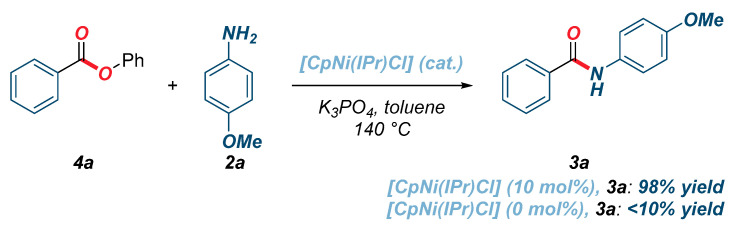
Amidation of activated phenolic ester using [CpNi(IPr)Cl].

**Scheme 2:**
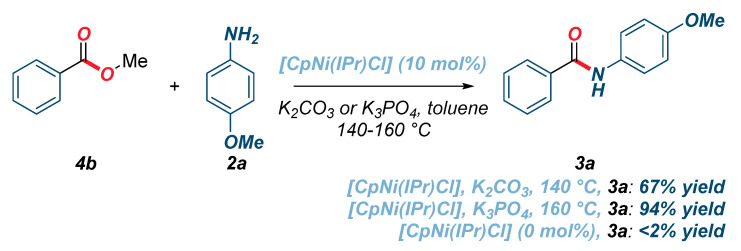
Amidation of unactivated methyl ester using [CpNi(IPr)Cl].

In consideration of the promising reactivity of twisted N-Boc amides using well-defined cyclopentadienyl half-sandwich [CpNi(IPr)Cl], we further explored amidation reactions of activated phenolic esters and unactivated methyl esters (Scheme 1 and Scheme 2). We were pleased to find that the amidation of phenyl benzoate proceeded in quantitative yield using K_3_PO_4_ as a base under otherwise the same reaction conditions as those used for the transamidation of amides (Scheme 1). Importantly, control reactions in the absence of the catalyst unambiguously verified that [CpNi(IPr)Cl] is required for the reaction. Interestingly, we also found that amidation of unactivated methyl benzoate proceeded in 67% yield, while a substantial enhancement of reactivity (94% yield) was observed by increasing the reaction temperature to 160 °C (Scheme 2). As expected, no reaction was observed in the absence of [CpNi(IPr)Cl] (<2%, not detected). 

**Figure 3:**
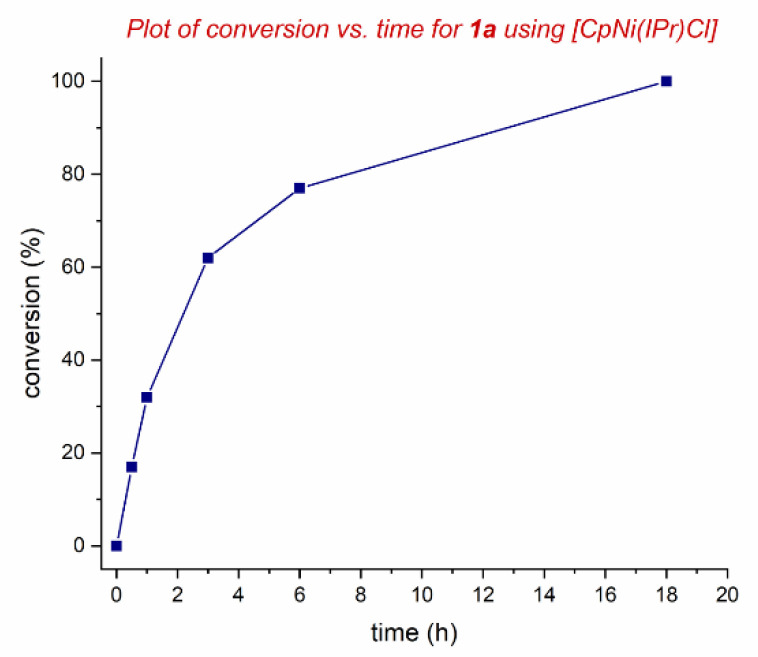
Kinetic profile of **1a**. Conditions: **1a**, 4-MeO-C_6_H_4_-NH_2_ (2.0 equiv), [CpNi(IPr)Cl] (10 mol%), K_2_CO_3_ (3.0 equiv), toluene (0.25 M), 140 °C, 0–18 h.

To gain preliminary insight into the reaction profile, kinetic studies were performed (Figure 3). As shown, the reaction reached 60% conversion after 3 h, while 77% conversion was observed after 6 h. The induction period was not observed in the kinetic profiling studies. We tentatively propose that the mechanism involves oxidative addition of the N–C bond to nickel. Other nickel sources, such as NiCp_2_ or NiCl_2_, catalyze the reaction albeit in lower yields. Studies on the mechanism and the expansion of the substrate scope are ongoing and will be reported in due course. 

## 3. Materials and Methods

General Information. General methods have been published [18].

General Procedure for [CpNi(IPr)Cl] Catalyzed Transamidation. In a typical procedure, an oven-dried vial was charged with a N-Boc amide or ester substrate (neat, 1.0 equiv), aniline (2.0 equiv), K_2_CO_3_ (3.0 equiv), [CpNi(IPr)Cl] (10 mol%), placed under a positive pressure of argon, and subjected to three evacuation/backfilling cycles under high vacuum. Toluene (0.25 M) was added at room temperature, the reaction was placed in a preheated oil bath at 140 °C, and stirred at 140 °C. After the indicated time, the reaction was cooled down, diluted with CH_2_Cl_2_ (10 mL), filtered, and concentrated. The sample was analyzed by ^1^H-NMR (CDCl_3_, 500 MHz) and GC-MS to obtain conversion, selectivity and yield using internal standard and comparison with authentic samples. All yields have been determined by ^1^H-NMR spectroscopy (CDCl_3_, 500 MHz). 

Representative Isolation Procedure for [CpNi(IPr)Cl] Catalyzed Transamidation. An oven-dried vial was charged with *tert*-butyl benzoyl(phenyl)carbamate (neat, 29.7 mg, 1.0 equiv), 4-methoxyaniline (24.6 mg, 2.0 equiv), K_2_CO_3_ (41.6 mg, 3.0 equiv), [CpNi(IPr)Cl] (10 mol%, 5.6 mg), placed under a positive pressure of argon, and subjected to three evacuation/backfilling cycles under high vacuum. Toluene (0.25 M) was added at room temperature, the reaction mixture was placed in a preheated oil bath at 140 °C, and stirred for 18 h at 140 °C. After the indicated time, the reaction was cooled down, diluted with CH_2_Cl_2_ (10 mL), filtered, and concentrated. A sample was analyzed by ^1^H-NMR (CDCl_3_, 500 MHz) and GC-MS to obtain conversion, yield and selectivity using internal standard and comparison with authentic samples. Purification by chromatography on silica gel (hexanes/ethyl acetate) afforded the title product. Yield 88% (20.1 mg). *N*-(4-Methoxyphenyl)benzamide. White solid. ^1^H-NMR (500 MHz, CDCl_3_) δ 7.86 (d, *J* = 7.5 Hz, 2 H), 7.76 (s, 1 H), 7.59–7.51 (m, 3 H), 7.47 (t, *J* = 7.4 Hz, 2 H), 6.91 (d, *J* = 8.9 Hz, 2 H), 3.81 (s, 3 H). ^13^C NMR (125 MHz, CDCl_3_) δ 157.00, 135.40, 132.04, 131.35, 129.10, 127.31, 122.43, 114.61, 55.86. [CpNi(IPr)Cl] has been prepared by the previously reported procedure [1].

## 4. Conclusions

In summary, we have reported on the transamidation reactions of *N*-activated amides by selective N–C(O) cleavage mediated by the well-defined, air- and moisture-stable half-sandwich [CpNi(IPr)Cl] complex. This class of Ni(II)–NHC cyclopentadienyl complexes has gained significant attention in organometallic catalysis owing to the beneficial properties of this class of catalysts; however, transamidation reactions of amides and amidation reactions of esters mediated by these complexes have been elusive. The present study demonstrates that highly selective transamidation of the N–C(O) bond in twisted N-Boc amides as well as activated phenolic and unactivated methyl esters with non-nucleophilic anilines under [CpNi(IPr)Cl] catalysis is feasible, thus providing an unconventional and unified method for the synthesis of secondary anilides by C(acyl)–N and C(acyl)–O bond cleavage reactions. It should be mentioned that the twisted amide starting materials are prepared from 2° amides by *N*-chemoselective *tert*-butoxycarbonylation [14], which provides a two-step transamidation method that could potentially be applied in late-stage derivatization of pharmaceuticals and natural products. The unique versatility of [CpNi(IPr)Cl] sets the stage for the broad application of Ni(II)–NHC cyclopentadienyl complexes in amide bond-forming reactions by N–C(O)/O–C(O) cleavage. Future studies will focus on the development of new classes of [CpNi(NHC)X] complexes for selective transformations of amide and ester bonds by N–C(O)/O–C(O) activation.

## Figures and Tables

**Table 1:**
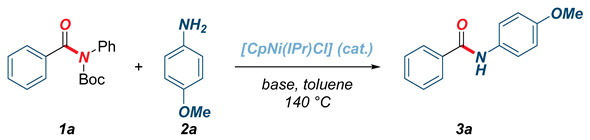
Optimization of the transamidation of amide **1a** using [CpNi(IPr)Cl].^1^

Entry	Catalyst	[Ni] (mol%)	Base	Solvent	Yield (%) ^2^
1	[CpNi(IPr)Cl]	10	K_2_CO_3_	toluene	>98
2	[CpNi(IPr)Cl]	10	K_3_PO_4_	toluene	>98
3	[CpNi(IPr)Cl]	5	K_2_CO_3_	toluene	74
4	[CpNi(IPr)Cl]	5	K_3_PO_4_	toluene	52
5	[CpNi(IPr)Cl]	-	K_2_CO_3_	toluene	<10
6	[CpNi(IPr)Cl]	-	K_3_PO_4_	toluene	<10

^1^ Conditions: amide (1.0 equiv), 4-MeO-C_6_H_4_-NH_2_ (2.0 equiv), base (3.0 equiv), [Ni] (0–10 mol%), toluene (0.25 M), 140 °C, 18 h. ^2^ Determined by ^1^H-NMR.

**Table 2:**
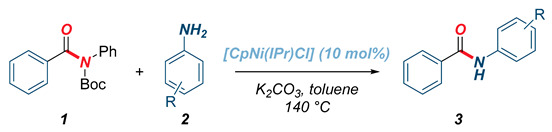
Scope of anilines in the transamidation of amide **1a** using [CpNi(IPr)Cl].^1^

Entry	Amide	Ar-NH_2_	3	Yield (%) ^2^
1	C_6_H_5_	4-MeO-C_6_H_4_	**3a**	98
2	C_6_H_5_	4-Me-C_6_H_4_	**3b**	97
3	C_6_H_5_	2-Me-C_6_H_4_	**3c**	77
4	C_6_H_5_	3,5-Me_2_-C_6_H_3_	**3d**	71
5	C_6_H_5_	4-F-C_6_H_4_	**3e**	64
6	C_6_H_5_	4-CF_3_-C_6_H_4_	**3f**	43

^1^ Conditions: amide (1.0 equiv), Ar-NH_2_ (2.0 equiv), K_2_CO_3_ (3.0 equiv), [CpNi(IPr)Cl] (10 mol%), toluene (0.25 M), 140 °C, 18 h. ^2^ Determined by ^1^H-NMR.

**Table 3:**
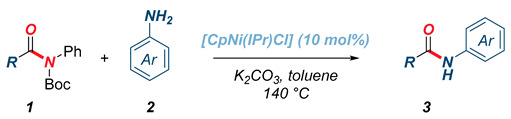
Scope of amides in transamidation with aniline **2a** using [CpNi(IPr)Cl].^1^

Entry	Amide	Ar-NH_2_	3	Yield (%) ^2^
1	*n*-C_9_H_19_	4-MeO-C_6_H_4_	**3g**	92
2	Cyclohexyl	4-MeO-C_6_H_4_	**3h**	98
3	4-Me-C_6_H_4_	4-MeO-C_6_H_4_	**3i**	86
4	4-MeO-C_6_H_4_	4-MeO-C_6_H_4_	**3j**	62
5	4-CF_3_-C_6_H_4_	4-MeO-C_6_H_4_	**3k**	62
6	Ph-CH=CH	4-MeO-C_6_H_4_	**3l**	73

^1^ Conditions: amide (1.0 equiv), Ar-NH_2_ (2.0 equiv), K_2_CO_3_ (3.0 equiv), [CpNi(IPr)Cl] (10 mol%), toluene (0.25 M), 140 °C, 18 h. ^2^ Determined by ^1^H-NMR.

## Sample Availability

Samples of the compounds are not available from the authors.
